# Promotion of Iron Oxide Reduction and Extracellular Electron Transfer in *Shewanella oneidensis* by DMSO

**DOI:** 10.1371/journal.pone.0078466

**Published:** 2013-11-07

**Authors:** Yuan-Yuan Cheng, Bing-Bing Li, Dao-Bo Li, Jie-Jie Chen, Wen-Wei Li, Zhong-Hua Tong, Chao Wu, Han-Qing Yu

**Affiliations:** 1 Department of Chemistry, University of Science & Technology of China, Hefei, China; 2 School of Life Sciences, University of Science & Technology of China, Hefei, China; Argonne National Laboratory, United States of America

## Abstract

The dissimilatory metal reducing bacterium *Shewanella oneidensis* MR-1, known for its capacity of reducing iron and manganese oxides, has great environmental impacts. The iron oxides reducing process is affected by the coexistence of alternative electron acceptors in the environment, while investigation into it is limited so far. In this work, the impact of dimethyl sulphoxide (DMSO), a ubiquitous chemical in marine environment, on the reduction of hydrous ferric oxide (HFO) by *S. oneidensis* MR-1 was investigated. Results show that DMSO promoted HFO reduction by both wild type and Δ*dmsE*, but had no effect on the HFO reduction by Δ*dmsB*, indicating that such a promotion was dependent on the DMSO respiration. With the DMSO dosing, the levels of extracellular flavins and *omcA* expression were significantly increased in WT and further increased in Δ*dmsE.* Bioelectrochemical analysis show that DMSO also promoted the extracellular electron transfer of WT and Δ*dmsE*. These results demonstrate that DMSO could stimulate the HFO reduction through metabolic and genetic regulation in *S. oneidensis* MR-1, rather than compete for electrons with HFO. This may provide a potential respiratory pathway to enhance the microbial electron flows for environmental and engineering applications.

## Introduction

*Shewanella oneidensis* MR-1 is a facultative anaerobic bacterium widely present in diverse environments [1], [2]. Under anaerobic conditions, it can utilize more than twenty electron acceptors including iron oxides. *S. oneidensis* MR-1 attracts great interest because it can reduce various toxic pollutants, such as organic pollutants, metals, metalloids, and radionuclides [3]–[5]. With increased knowledge on its respiration in recent years, *S. oneidensis* MR-1 has been frequently used as a model microorganism to study the roles of dissimilatory metal reducing bacteria in biogeochemical cycling and bioremediation or bioenergy production application [6]–[8].

Dissimilatory reduction of iron oxides by *S. oneidensis* MR-1 is of environmental significance. Such a process is coupled with the oxidation of organic matters, and affects geochemical cycling of both carbon and iron. Reduction and consequent dissolution of iron oxides can result in the release of phosphate, trace metals and even contaminants absorbed by iron oxides [9], [10]. In addition, *S. oneidensis* MR-1 indirectly affect pollutant transformation through producing Fe(II) which is able to reduce some pollutants directly [11], [12]. For these reasons, impacts of iron oxide reduction by *S. oneidensis* MR-1 on redox cycling in subsurface, chemical migration and pollutant degradation have been studied for decades. *S. oneidensis* MR-1 reduces iron oxides through a typical extracellular electron transfer (EET) process, in which electrons derived from substrate oxidation are transferred to electron acceptors outside cells. EET is crucial for many microbial reduction processes and applications, ranging from syntrophic coculturing to element geochemical cycling, bioremediation and electricity generation [13]–[15]. The EET capability of *S. oneidensis* MR-1 depends strongly on flavins and some cell surface c-type cytochromes (c-Cyts) including OmcA and MtrC. Flavins, a type of electroactive metabolites synthesized and secreted by many *Shewanella* species, can assist EET by shuttling electrons from cell surface to iron oxides or anodes in bioelectrochemical systems [16]. Flavins can contribute to ∼75% of electron transfer by *S. oneidensis* MR-1 for current generated in electrochemical cells [17]. Dose of flavins at a micromole level increases current by about 5-folds in microbial fuel cells [18]. *S. oneidensis* MR-1 encodes *ribBA* and *ribB* which are homologs in *Bacillus* and *Escherichia coli*, respectively, for the synthesis of riboflavin, the precursor for synthesis of other flavins [19]. Despite of the important role of flavins in EET, regulations on their synthesis and secretion *S. oneidensis* MR-1 are largely unclear yet.

Another key component for EET and iron oxides reduction is the c-Cyts, especially those anchored at cell surface [20]. OmcA and MtrC are two essential cell-surface c-Cyts responsible for electrons transfer to iron oxides [21]. Lack of these c-Cyts would result in a great decrease in iron oxide reduction [22]. Electrochemical analysis has also confirmed the direct electron transfer from OmcA and MtrC to hematite electrodes [23]. Moreover, it has been revealed that both OmcA and MtrC play a critical role in many other EET-dependent reduction processes, including extracellular reduction of Cr(VI) and U(VI) [5], [24]. CymA, as a c-Cyt anchored in the cytoplasmic membrane and faced to periplasm, is the hub of electron transfer pathways for anaerobic respiration of *S. oneidensis* MR-1 [25], [26]. Fluctuation in the level of those biological components inevitably influences the hydrous ferric oxide (HFO) reduction and EET, while information about such processes at the coexistence of electron acceptors has been limited so far.

Coexistence of multiple electron acceptors is commonly encountered in diverse environments. DMSO, one of electron acceptors used by *S. oneidensis* MR-1, is a methylated sulfur compound and commonly present in marine environments. The reducing product of DMSO by *S. oneidensis* MR-1 is volatile dimethyl sulfide (DMS), which plays a role in the global radiation balance, thereby suggesting the environmental relevance of microbial DMSO respiration [27]. Despite of its high solubility, DMSO is used as an extracellular electron acceptor by *S. oneidensis* MR-1 [28]. DMSO reductase subunits in *S. oneidensis* MR-1 encoded by *dmsEFABGH* operon. DmsE is a periplasmic c-Cyt transferring electrons from CymA to DMSO terminal reductase DmsAB which are localized on the outer surface of outer membrane. *dmsE* mutant (Δ*dmsE*) showed impaired growth on the DMSO but a greatly increased DMSO reductase activity. *dmsB* mutant (Δ*dmsB*) lost its ability to grow on DMSO. Therefore, DMSO reduction by *S. oneidensis* MR-1 shows a similarity with HFO reduction in terms of EET and such a similarity suggests a possible competition of DMSO respiration with iron oxides reduction and other EET processes for electrons.

Therefore, this work aims to explore the effects of DMSO on HFO reduction by *S. oneidensis* MR-1. Chemical, biological, bioelectrochemical and computational analyses were conducted to evaluate the possible effects and to reveal the underlying mechanism. Results from this study should contribute to a better understanding about the iron oxide reduction by *S. oneidensis* MR-1 and provide useful information to manipulate the EET of *S. oneidensis* MR-1 for bioremediation and bioenergy generation.

## Materials and Methods

### Growth Conditions

*S. oneidensis* MR-1 wild type (WT) and the mutant strains were pre-cultured aerobically in Luria–Bertani (LB) medium at 30°C for 16 h until stationary phase. For HFO reduction, the culture was collected by centrifugation and washed using basal medium (BM) [4] for three times. Concentrated cultures were injected into sealed serum vials containing 30 ml anaerobic BM to a final concentration of 2.0 in OD_600_. The anaerobic BM was supplemented with 50 mM lactate as the electron donor and carbon source. The concentration of HFO and DMSO was 20 mM, unless indicated otherwise.

### Mutant Strain Construction

Mutants with an in-frame deletion of the desired genes were constructed essentially as described previously [29]. Brifely, chimeric DNA fragments with flanking regions of target genes were amplified and ligated by PCR using primers F1/R1/F2/R2. Then DNA fragment was digested and ligated with pRE112. The resulting plasmids were transformed into *E. coli* WM3064 and subsequently introduced into *S. oneidensis* MR-1 through conjugation. After two rounds of selection, mutants deleted of target genes were confirmed by PCR using primer pairs (CheckF/R) upstream and downstream of the location of the chimeric DNA fragments. Primers used for mutant construction are listed in Table 1.

**Table 1:** Strains, plasmids and primers used in this study.

Strain	Relevant genotype or phenotype	Source or reference
***S. oneidensis***		
MR-1	wild-type	
Δ*dmsE*	mutant with deletion of SO1427 in in *S. oneidensis* MR-1	22
Δ*dmsB*	mutant with in-flame deletion of SO1430 in *S. oneidensis* MR-1	This study
***E.coli***		
JM109	F′ *traD36 proA^+^B^+^ lacI^q^ Δ(lacZ)M15/Δ(lac-proAB) glnV44 e14^−^ gyrA96 recA1 relA1 endA1 thi hsdR17*	Lab stock
WM3064	*thrB1004 pro thi rpsL hsdS lacZ*ΔM15 RP4-1360Δ *(araBAD)567*Δ*dapA1341::*[erm pir(wt)]	Lab stock
**Primer**		
RT-*ribB*	F-TGGTCATACCGAAGGCACTATR-CAGGGCACCAAAGGCGATA	This study
RT-*ribBA*	F-TTGAACGAAGACGGCACTATGR-TTGGCTTCACGCACAACG	This study
RT-*omcA*	F-GATACTCGCTACGCTTACATCCR-TCCTTGTTATGGCAACCTGAAC	This study
RT-*mtrC*	F-CGGCAATGATGGTAGTGATGGR-TGGCATGTCGGCTTCGTTA	This study
RT-*cymA*	F-GGTTGTTGGTATCGTGATTGGTR-GCAGATGCCAGCACTTCATT	This study
Del-*dmsB* a,b	F1-TCCGAGCTCAGGCTACGACGATGAAGATACTR1-CCTTACGCTCGATATCCAG*CGGTCCTTACAGGCAATATG*F2-ATTGCCTGTAAGGACCG*CTGGATATCGAGCGTAAGGTGAT*R2-CGGGGTACCATCGTCCTGCCATTGAGTCACheckF-AGGCTACGACGATGAAGATACTChenkR-ATTGGTGACTGACGCTAATGAC	This study

a Underline indicates the recognition sites by endonuclease.

b Italic indicates the annealing sites with downstram/upstream primers.

### Synthesis of Hydrous Ferric Oxide and Fe (II) Assay

The HFO was prepared essentially following the method reported previously [22]. Briefly, the HFO was synthesized by neutralizing a 0.4 M FeCl_3_ solution to pH 7.0 through dosing NaOH solution under stirring. The HFO was collected by centrifugation (30 min, 2100×g, 20°C), then washed with deionized water for six times to remove chloridion and finally lyophilized before use. The reduction of HFO was evaluated from the formed Fe(II) concentration using ferrozine assay [30].

### Extracellular Flavins Analysis

The concentrations of riboflavin-5′-phosphate (FMN) and riboflavin were determined following a previously-reported method [16]. Briefly, 50 µL samples were injected into a liquid chromatography (LC-1100, Agilent Inc., USA) equipped with 4.6 mm×150 mm Symmetry C18 column with a 5 µm particle size (Waters Inc., Ireland). The mobile phase consisted of 25% methanol, 75% ammonium acetate (0.05 M, pH 7.0) in deionized water at a flow rate of 0.8 ml/min. The column was maintained at 30°C. Flavins were detected with an RF-10AXL fluorescence detector (Shimadzu Co., Japan) at an excitation wavelength of 420 nm and an emission wavelength of 525 nm. FMN and riboflavin (Sigma Inc., USA) standard solutions were prepared in BM at concentrations ranging from 0.1 to 10 mM. Concentrations of FMN and riboflavin were calculated by comparing the integrated area of each peak to the area of standard peaks.

### DMSO Analysis

The samples were centrifugated at 12,000×g for 5 min, and then 20 µl of the supernatants were taken for analysis by a high performance liquid chromatography system (HPLC1200, Agilent, USA) equipped with a diode array detector set at 210 nm. Separation was achieved on an Aminex HPX-87H column (300 mm×7.8 mm, 9 µm particle size, Bio-Rad, USA). 0.005 M H_2_SO_4_ was used as eluent with a flow rate of 0.5 ml/min. The column temperature was set at 50°C. Commercially available DMSO (Sangon Co., China) was used to obtain a calibration curve to access the concentration of DMSO.

### RNA Extraction and qRT-PCR

Total cellular RNA from cultures was extracted using RNAiso Plus (Takara Co., China). The concentration and purity of the final extracted RNA were determined according to absorption of light at 230, 260 and 280 nm. The cDNA was synthesized using PrimeScript II 1st Strand cDNA Synthesis Kit and the qRT-PCR was performed using the SYBR Premix Ex Taq (Takara Co., China) according to manufacturer’s instruction. The qRT-PCR was run in a StepOne real-time PCR system (Applied Biosystems Inc., USA). The relative quantity of cDNA normalized to the abundance of 16S cDNA was automatically calculated by the StepOne real-time PCR system. Primers used for qRT-PCR analysis were listed in Table 1.

### Electrochemical Analysis

Electrochemical measurements were conducted with a CHI1030A electrochemical workstation (CH Instruments Co., China). Cells were incubated in one chamber electrochemical cell of 150 ml. A square indium tin oxide glass of 25 mm side length was used as the working electrode. A platinum wire and a saturated Ag/AgCl electrode were used as counter and reference electrode, respectively. BM with 50 mM lactate was used as electrolyte solution, and 100 ml of solution was fed in the electrochemical cell. The working electrode was poised at +0.15 V (vs. Ag/AgCl). Cultures grown overnight in LB medium were collected and washed three times using BM, and were then inoculated into electrochemical cells under anaerobic condition. The electrochemical cells were gently stirred and incubated at 30°C.

### Computational Methods

The structure of the HFO coordination compounds with H_2_O and DMSO are studied by density functional theory (DFT) computation. In the calculation, an all-electron method, which is within the Perdew, Burke, and Ernzerhof forms of generalized gradient approximation [31] for the exchange-correlation term, is used as implemented in the DMol^3^ code [32], [33]. This method adopts double precision numerical basis sets that take into account p polarization on all atoms. The energy in each geometry optimization cycle is converged to within 1×10^−5^ Hartree with a maximum displacement and force of 5×10^−3^ Å and 2×10^−3^ Hartree/Å, respectively.

## Results

### DMSO Enhances HFO Reduction by *S. Oneidensis* MR-1

**Figure 1:**
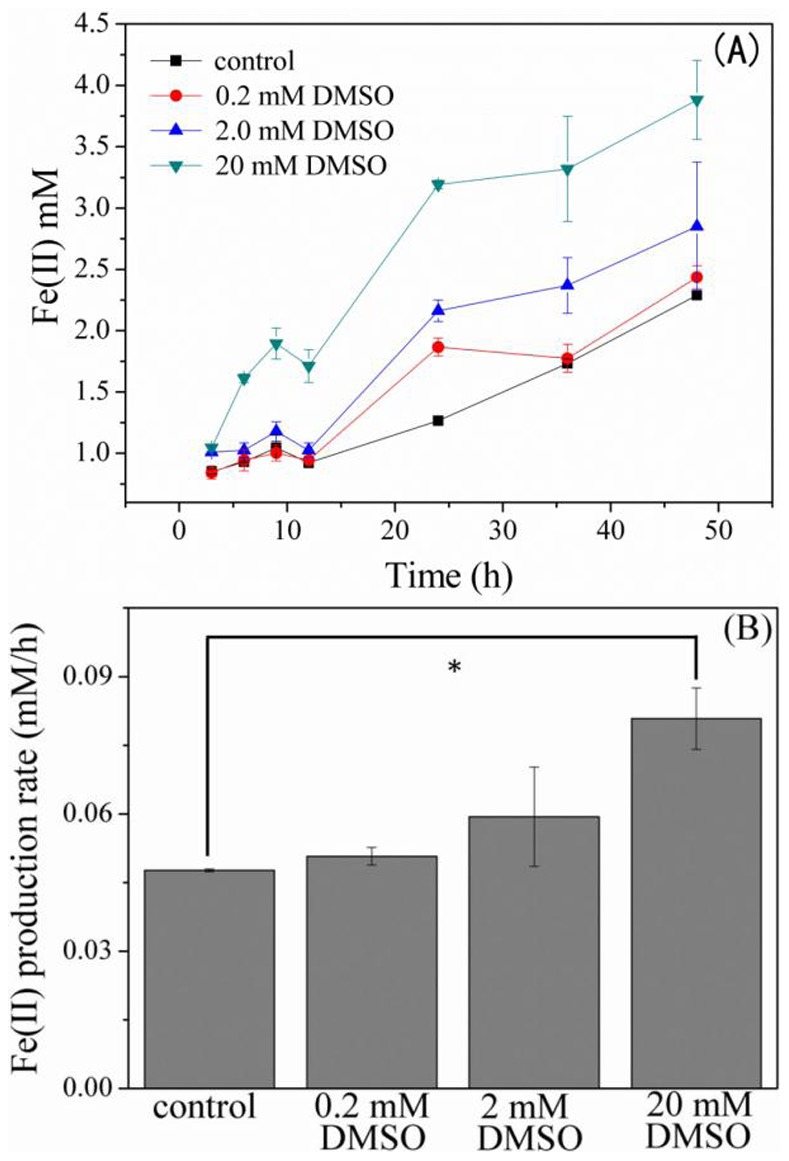
HFO reduction in the presence of DMSO. (A) HFO reduction *by S. oneidensis* MR-1 with different concentration of DMSO. (B) Fe(II) producing rate after 48 hours reduction of HFO. Resting cells were incubated with 20 mM HFO and DMSO with various concentrations. The data are average of triple samples and error bars represent standard deviation. Asterisk indicates significant difference, *p*<0.05.

To investigate the impact of DMSO on the HFO reduction by *S. oneidensis* MR-1, concentrations of the formed Fe(II) at various DMSO dosages were measured. For the HFO reduction, resting cells, which were in their stationary phase with negligible growth, were used to minimize the microbial growth during the testing period. HFO reduction showed a dose-dependent response to DMSO and the fastest reduction was observed when 20 mM DMSO was dosed (Figure 1A). Dose of 20 mM DMSO significantly increased the HFO reduction rate (Figure 1B), indicating an obvious promoting effect of DMSO on HFO reduction. The possible bacterial growth, which consequently affects the HFO reduction rate, was evaluated by measuring the total proteins concentration. The result shows that there was no significant difference in biomass amount in the presence and absence of DMSO during the testing time period (Supporting Information, Figure S1). To determine whether DMSO can change the solubility of HFO and consequent HFO reduction by *S. oneidensis* MR-1, the DFT computation has been performed to identify the structures of the coordination compounds. The ligand exchange for high-spin Fe(III) (*t*_2*g*_^3^ *e_g_* ^2^) occurs with a higher rate constant (*k*_ex_) for H_2_O (1.6×10^2^ s^−1^) than for DMSO (0.93×10^1^ s^−1^) through an associative interchange mechanism [34], [35], indicating that Fe(III) has larger affinity to ligand H_2_O than DMSO. Moreover, three ligands of H_2_O or DMSO coordinated with HFO on the iron ion have been calculated by the DFT method (Figure S2). This thermodynamic properties analysis reveals that the FeO(OH) coordinated by the ligand of H_2_O molecules has more negative Gibbs free energy (−7.903 kcal/mol) than DMSO does (−1.344 kcal/mol), indicating that the Fe(III) could be more favorable to coordinate with the H_2_O molecules than DMSO ligand. Taken together, these results clearly demonstrate that the presence of DMSO enhanced the HFO reduction by *S*. *oneidensis* MR-1 through promoting the HFO respiratory activity.

### DMSO Respiration Affects HFO Reduction

**Figure 2:**
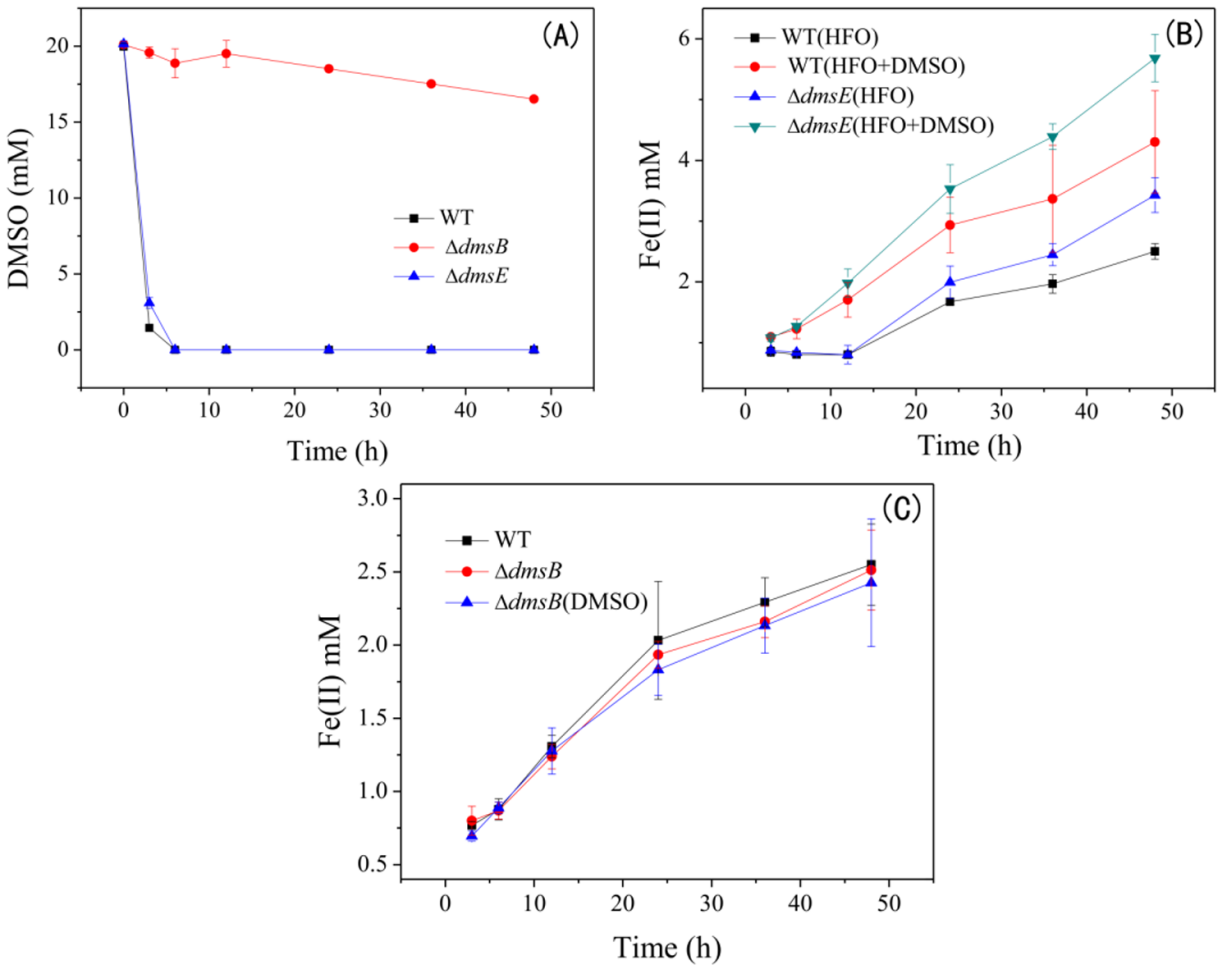
DMSO reduction by WT, Δ*dmsE* and Δ*dmsB* (A). HFO reduction by Δ*dmsE* (B) and Δ*dmsB* (C) compared with WT with dose of 20 mM DMSO. The data are the average of triple samples and the error bars represent standard deviation.

So far, there has been no direct evidence for the DMSO reduction by mutants of DMSO reductases. Thus, we initially tested their DMSO reduction capacities. Δ*dmsE* showed no obvious defect in DMSO reduction, while Δ*dmsB* completely lost the DMSO reduction ability (Figure 2A). HPLC analysis showed that the decrease in DMSO was quantitatively correlated with the increase of DMS (Figure S3). These results directly indicate that, under the resting conditions, DMSO respiration of Δ*dmsE* was comparable with WT, while Δ*dmsB* completely lost the DMSO respiration ability. Then, the HFO reduction by Δ*dmsE* and Δ*dmsB* was compared to evaluate the effect of DMSO respiration on HFO reduction. Δ*dmsE* showed a significantly enhanced HFO reduction compared with WT no matter in the absence or presence of DMSO (Figure 2B). Abiotic reduction of HFO by DMS and possible effect of DMS on bioreduction of HFO by *S. oneidensis* MR-1 was eliminated (Figure S4). Since Δ*dmsE* has increased DmsAB expression and DMSO reductase activity [28], it was postulated that DmsAB, the terminal reductase complex of DMSO, might contribute to the HFO reduction. However, Δ*dmsB* showed a comparable HFO reduction with WT in the absence or presence of DMSO (Figure 2C). Thus, the participation of DmsB in HFO reduction could be excluded. On the other hand, DMSO stimulated HFO reduction by Δ*dmsE*, but had no effects on Δ*dmsB*, suggesting that the capability for DMSO respiration might be the trigger for promoting the HFO reduction.

### DMSO Improves Flavins Secretion in *S*. *Oneidensis* MR-1

**Figure 3:**
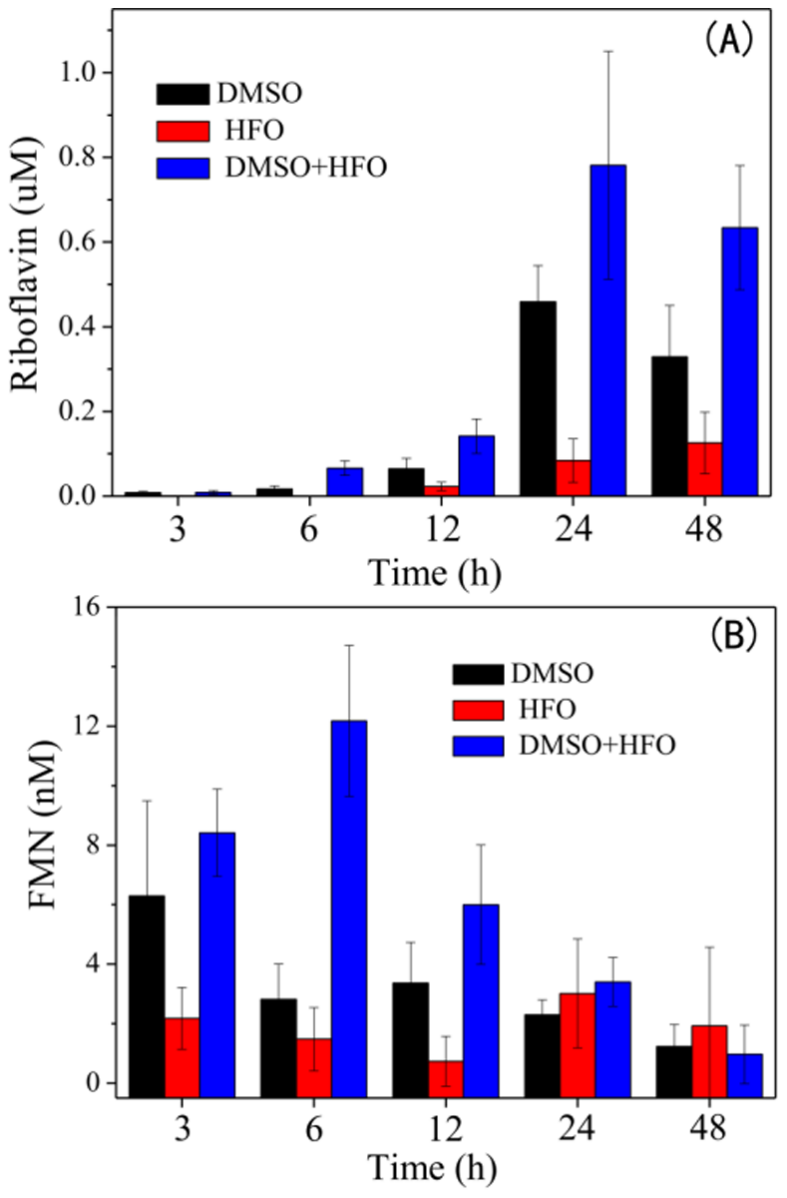
Flavins secretion by *S. oneidensis* MR-1. Concentration of riboflavin (A) and FMN (B) secreted by *S. oneidensis* MR-1 in the presence of 20 mM HFO, or 20 mM DMSO or both. The data are the average of triple samples and the error bars represent standard deviation. Concentration of formed Fe(II) through HFO reduction by *S. oneidensis* MR-1 wild type (WT) was compared with that by the Δ*dmsE* mutant (A), and with the *dmsB* mutant (B).

Secretion of flavins is a unique property for many *Shewanella* species, that distinguishes them from many other dissimilatory metal-reducing bacteria [19]. Thus, levels of flavins were measured in HFO reduction in the absence and presence of DMSO. Flavins secreted by *S. oneidensis* MR-1 include flavin mononucleotide (FMN) and riboflavin. As illustrated in Figure 3, DMSO, as the sole electron acceptor, significantly increased the secretion of extracellular riboflavin and FMN by WT, compared without dose of DMSO. The highest levels of riboflavin and FMN were found with the coexistence of DMSO and HFO. The riboflavin level gradually increased over time and reached sub-micromole level. Unlike riboflavin, the FMN concentration quickly increased and reached a plateau of nanomole level after 6 h and then gradually decreased. This observation suggests that riboflavin was the major electron shuttle secreted by *S. oneidensis* MR-1 under the testing conditions.

### Transcriptional Alteration of Genes Involved in HFO Reduction

**Figure 4:**
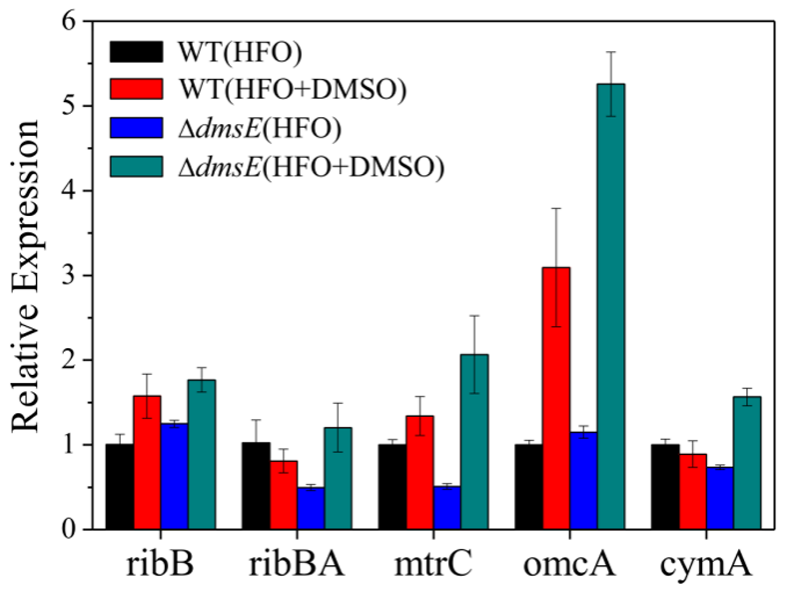
Expression of *cymA, mtrC, omcA, ribB, ribBA* at 6-h in HFO reduction by WT and Δ*dmsE* with or without 20 mM DMSO. The error bars indicate the standard deviation.

The transcription of genes presumably involved in riboflavin biosynthesis was tested. Without DMSO, the expression of *ribBA* and *ribB* after the 6 h HFO reduction was detected using qRT-PCR. Transcription of *ribB* and *ribBA* is distinguishingly altered for the presence of DMSO while none of alteration is large than 1-folds (Figure 4). Besides, the expression of OmcA and MtrC was evaluated in the HFO reduction in the presence of DMSO. With DMSO dosing, expression of OmcA was increased about 3-fold and 4.5-fold in WT and Δ*dmsE*, respectively. MtrC expression was increased about 4-fold in Δ*dmsE*, but only showed a slight increase in WT (Figure 4). The level of OmcA and MtrC was the highest in Δ*dmsE* with DMSO dosing, which is consistent with the HFO reduction results. Given the role of OmcA and MtrC in EET, this results suggest that the increased expression of OmcA and MtrC contributed to, at least partially, the enhanced HFO reduction. Furthermore, the expression of CymA, the hub controlling anaerobic respiration of *S. oneidensis* MR-1, was also monitored. Dose of DMSO caused a minor decrease in the expression of CymA in WT (Figure 4). It seems that the CymA level was not the limiting factor for the DMSO-promoted HFO reduction in resting cells. The level of CymA was significantly increased in Δ*dmsE* with DMSO dosing, which is also consistent with the HFO reduction result.

### DMSO Enhances the EET from *S. Oneidensis* MR-1 to Solid Electrode

**Figure 5:**
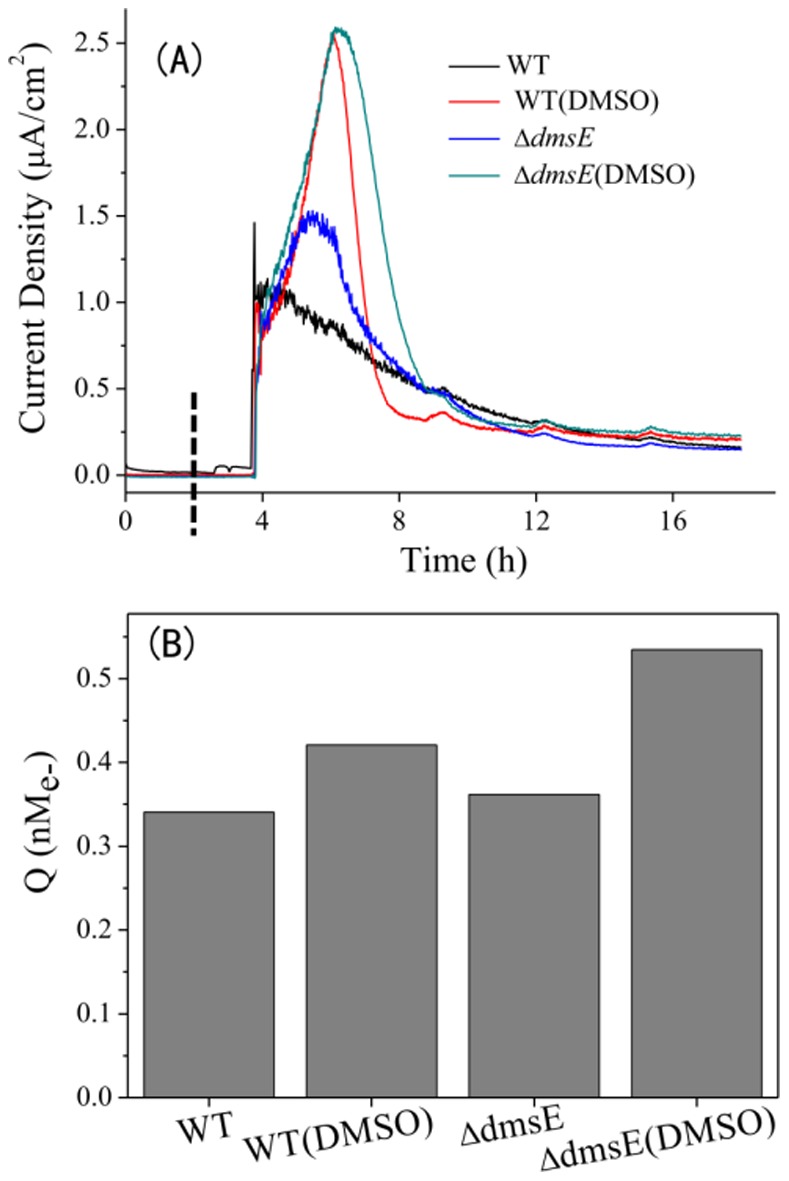
Electricity currents generation in bioelectrochemical system. Current generated by WT and the Δ*dmsE* in electrochemical cells (A) and the coulomb integral of each electrochemical cell across the test period (B). The potential of working electrodes was set at +0.15 V (versus Ag/AgCl). DMSO was dosed at 0.5 mM. Microbial cells were inoculated to the OD600 of 0.5 at given time point, which is indicated by dotted line. The experiment was conducted three times and consistent results were obtained.

In addition to HFO reduction, electron transfer from microbial cells to solid electrodes was also examined to further evaluate the impact of DMSO on the EET of *S. oneidensis* MR-1. For this purpose, the bio-electricity current generated in three-electrode electrochemical cells posed at +0.15V (vs. Ag/AgCl) was monitored. The current density produced by the Δ*dmsE* dosed with 0.5 mM DMSO reached an average of 2.49 µA/cm^2^, while that by WT only produced 1.00 µA/cm^2^ in the absence of DMSO (Figure 5A). The amount of electrons (Q) transferred to electrodes was calculated by integration of the current. This result suggests that the Δ*dmsE* in the presence of DMSO transferred most electrons to the electrode (Figure 5B). The abiotic reduction of electrode by DMS or DMSO was excluded (Figure S5). Thus, these results indicated that DMSO not only stimulated the HFO reduction by *S. oneidensis* MR-1, but also enhanced the bacterial EET from cells to the electrode in bioelectrochemical systems.

## Discussion

This study demonstrates that DMSO does not suppress the HFO reduction by *S. oneidensis* MR-1 despite of the possible competition for electrons. Instead, DMSO enhances the HFO reduction and current generation. Δ*dmsB*, a mutant unable to reduce DMSO, shows no response to DMSO in the HFO reduction, indicating that the promotion of HFO reduction is dependent on DMSO respiration. This result is possibly attributed to the fact that reducing DMSO boosts anaerobic respiration, which contributes to EET for HFO reduction and current generation. Previous studies have shown that power output of microbial fuel cells inoculated with *Shewanella* species was promoted by oxygen, a favor electron acceptor for *Shewanella* species [36]. Li et al. proposed that oxygen induced a higher overall metabolic rate, which is responsible for the increased current generation [37]. In our study, 20 mM DMSO is quickly reduced by WT after 6 hours (Figure 2A), which might cause a higher metabolic rate contributing to the HFO respiration. Δ*dmsE* shows a growth defect on DMSO [28], but has comparable DMSO reduction with WT under resting state (Figure 2A). A promotion effect of DMSO on HFO reduction is also observed for Δ*dmsE*. These results indicate a DMSO-respiration-dependent promotion of HFO reduction, which might result from the promoted anaerobic respiration. Besides, transcriptional profile analysis shows that transcription of some genes involved in metal reduction, energy metabolism was differentially regulated upon being exposed to different electron acceptors including DMSO [38]. Thus, a cascade of transcriptional events might be respond to DMSO respiration.

Moreover, Δ*dmsE* shows an obviously enhanced HFO reduction than WT even in the absence of DMSO. Inconsistent results have been reported in previous studies about whether DmsE contributes to HFO reduction [22], [39]. Considering the low reduction rate of HFO, the high cell density in both Bretschger’s report [22] and our study might be the reason for accentuating the role of DmsE in HFO reduction during a short time period. DmsE is localized in the periplasmic space to transfer electrons from CymA to DmsAB. In addition to interacting with DmsE, CymA can also transfer electrons to many other c-Cyts in periplasmic space, such as *mtrA* (for ferric reduction), *fccA* (for fumarate reduction), *napB* (for nitrate reduction), *nrfA* (for nitrite reduction) and *cctA* [26], [39], [40]. These c-Cyts, together with CymA, construct an electron transfer network in periplasm to readily transport electrons to diverse electron acceptors available. CymA seems to have weak and transient interactions with these c-Cyts in periplasm and can be easily separated and purified [41]. DmsE is the homologue of MtrA and phylogenetic analysis implies that DmsE might evolve from MtrA which is also located in the periplasm and is essential for the reduction of iron oxides. DmsE facilitates electron transfer to outer-membrane cytochromes in the absence of MtrA and MtrD [39]. It displays a lower macroscopic potential than MtrA and overlapped potential window with that of CymA [42]. These results suggest that DmsE is a potential competitor with MtrA for binding sites of CymA and electrons, thereby limiting electron transfer to MtrA and consequent HFO reduction. The increased HFO reduction by Δ*dmsE* probably results from the vanished competition. The Mtr pathway, including CymA-MtrA-OmcB-OmcA/MtrC, is not only essential for HFO reduction, but also is broadly involved in the EET-dependent reduction of many compounds. Thus, Δ*dmsE* might have better EET performance when Mtr pathway is involved. Indeed, Δ*dmsE* generates the highest electrical current (Figure 5).

Levels of flavins and OmcA/MtrC are examined for their essential roles in EET. Flavins are self-produced and secreted electron shuttles by most *Shewanella* species. *S. oneidensis* MR-1 possesses two sets of genes which presumably involved in riboflavin biosynthesis [19], while no experimental evidence has been presented so far to examine the physiological function of these genes. The transcription analysis in this study shows that the expression of *ribB* and *ribBA* was only slightly altered at 6 h when the 20 mM DMSO was completely consumed, suggesting that increased levels of extracellular flavins might not primarily contributed to the expression of both genes at that time. Except for synthesis, levels of extracellular flavins can also be affected by the process of secretion. A recent study reported a protein essential for flavins secretion by *S. oneidensis* MR-1 [43]. This protein belongs to the multidrug and toxin efflux transporter family, a homolog in which family functions in an energy-dependent manner in *Brucella melitensis* [44]. The possible energy requirement for flavins secretion is likely to be facilitated by respiration on readily available electron acceptors, such as DMSO. Besides, it was also found that dose of DMSO stimulated the complete oxidation of lactate to acetate (Figure S6), which partially supports the proposed mechanism of energy generation.

Expression of OmcA and MtrC are also differentially regulated. Dose of DMSO results in a 2-fold increase in OmcA expression by WT, and more than 4-fold and 1-fold increases in OmcA and MtrC expression by Δ*dmsE*, respectively. OmcA and MtrC play a great role in many EET-dependent reduction processes, such as extracellular reduction and precipitation of uranium and chromium [5], [24]. Their expression in *E. coli* can promote the HFO reduction by more than 100% [45]. CymA, as the hub of anaerobic respiration, is constitutively expressed when diverse electron acceptors are used [38], [46]. Consistently, dose of DMSO has no significant effect on the CymA expression in WT and Δ*dmsB* (Figure 4). However, CymA expression is significantly increased in Δ*dmsE* with DMSO. CymA, as a quinol dehydrogenase, is considered to harness the electrons exported from quinol-pool during anaerobic respiration. Besides, it may contribute to the generation of proton-motive force as part of a redox loop [26]. Thus, an increased level of CymA is likely to promote electron transport and even energy production from proton-motive force generation, which might be one of reasons for the obviously promoted HFO reduction in Δ*dmsE* with DMSO dosing (Figure 2B). A previous study has reported that transcription of *dmsAB* was increased by 4.5-fold in Δ*dmsE* [28], indicating that deletion of *dmsE* can cause transcriptional regulation with an unidentified mechanism. Both genetic and metabolic mechanisms might have contribution to the greatly promoted EET of Δ*dmsE* in the presence of DMSO.

## Supporting Information

Figure S1 **Cell growth during HFO reduction by** ***S oneidensis*** **MR-1 at different DMSO concentrations.** Aliquots of cultures for HFO reduction were centrifuged to collect cells. The pelleted cells were resuspended in 500 µL lysis buffer (50 mM Tris-Cl, 1 mM EDTA, 200 mM NaCl, 0.5% Triton X-100, 1 mM PMSF). Cells were lyzed by ultrasonic lysis treatment for 90 times (one time includes a treatment for 3 s and an interval of 3 s). Total concentration of proteins was determined by the bicinchoninic acid (BCA) assay using BCA protein assay kit (Sangon Co., China).
(DOCX)

Figure S2 The coordination structures of FeO(OH) with three ligands of H_2_O (a) and DMSO (b). Carbon atoms colored in gray, hydrogen white, oxygen red, sulfur yellow, and iron purple.
(DOCX)

Figure S3 **Reduction of DMSO and production of DMS by** ***S. oneidensis*** **MR-1 strains.** DMSO was dosed at 2 mM for reduction. Samples at indicated time points from liquid and gaseous phases were collected for DMSO and DMS analysis respectively. DMS concentration was measured using a gas chromatographysystem (GC7890A, Agilent Co., USA) with a flame ionization detector. Gas was sampled from headspace of serum bottles with a syringe and directly injected into gas chromatography for analysis. A commercial GC capillary column (DB-FFAP, 30 m×0.25 mm×0.25 µm, J&W Scientific Inc., USA) was used for separation. Nitrogen (99.999%) was used as the carrier gas. The temperatures of the injector and detector were set at 250°C and 300°C, respectively. The oven temperature profile was programmed as follows: 70°C held for 3 min and ramped to 200°C at 20°C/min held for 3 min.
(DOCX)

Figure S4 **Effect of DMS on HFO reduction by** ***S oneidensis*** **MR-1.** 20 mM DMS was dosed into serum vials in HFO reduction. Control was set as no cells to evaluate possible abiotic reduction of HFO by DMS.
(DOCX)

Figure S5 **Effect of DMS on electricity generated by the WT.** Bacterial cells were inoculated to the OD_600_ of 0.5. The potential of working electrodes was set at +0.15 V (versus Ag/AgCl). DMSO and DMS were dosed at 0.5 mM. Electrochemical cells dosing DMSO or DMS and without cultures were set as controls. The experiments were repeated twice.
(DOCX)

Figure S6 **Lactate consumption and acetate production at 48 h of HFO reduction.** The medium was supplemented with 50 mM lactate for HFO reduction. 20 mM DMSO was also added when indicated.
(DOCX)
