# Supplementary material for: Promotion of Iron Oxide Reduction and Extracellular Electron Transfer in Shewanella oneidensis by DMSO
Source: PLoS One. 2013 Nov 7;8(11):e78466. doi: 10.1371/journal.pone.0078466 (PMC3820605; doi:10.1371/journal.pone.0078466)
Supplement: Figure S1 — Cell growth during HFO reduction by S oneidensis MR-1 at different DMSO concentrations. Aliquots of cultures for HFO reduction were centrifuged to collect cells. The pelleted cells were resuspended in 500 µL lysis buffer (50 mM Tris-Cl, 1 mM EDTA, 200 mM NaCl, 0.5% Triton X-100, 1 mM PMSF). Cells were lyzed by ultrasonic lysis treatment for 90 times (one time includes a treatment for 3 s and an interval of 3 s). Total concentration of proteins was determined by the bicinchoninic acid (BCA) assay using BCA protein assay kit (Sangon Co., China). (DOCX) [file pone.0078466.s001.docx]

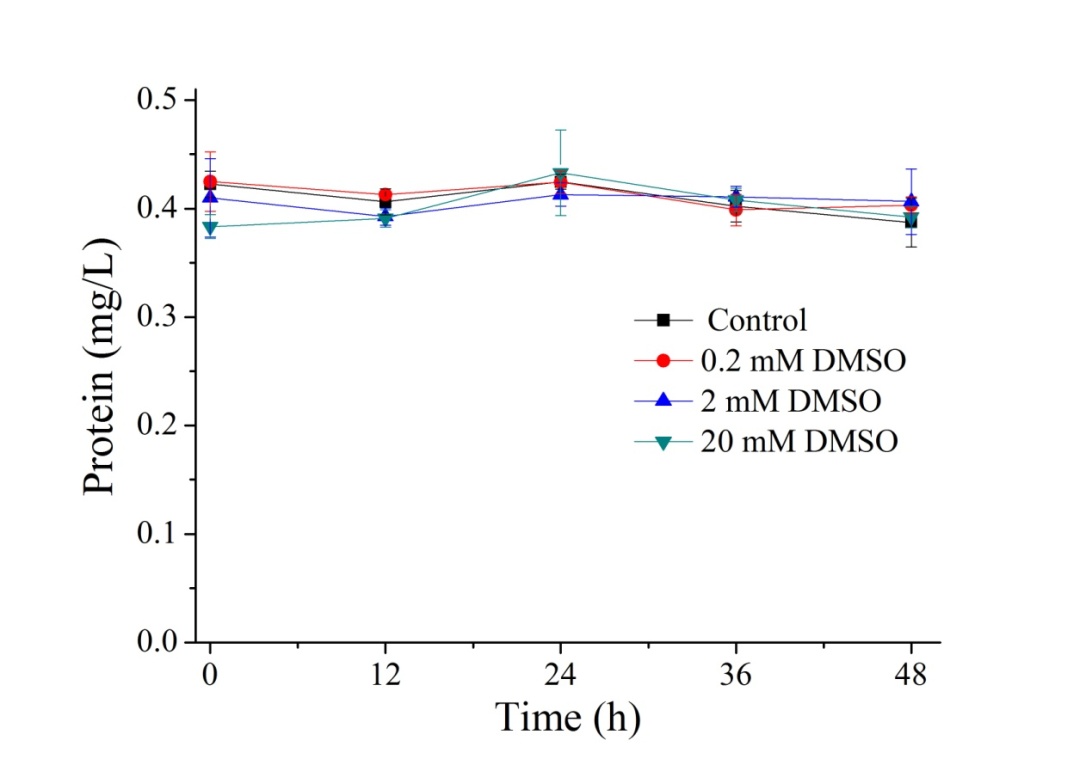


**Figure S1. Growth during HFO reduction by *S oneidensis* MR-1 under different DMSO concentration.** Aliquots of cultures for HFO reduction were centrifuged to collect cells. The pelleted cells were resuspended in 500 μL lysis buffer (50 mM Tris-Cl, 1 mM EDTA, 200 mM NaCl, 0.5% Triton X-100, 1 mM PMSF). Cells were lyzed by ultrasonic lysis treatment for 90 times (one time includes a treatment for 3 s and an interval of 3 s). Concentrations of total protein were determined by the bicinchoninic acid (BCA) assay [[1](#_ENREF_1)] using BCA protein assay kit (Sangon Co., China).

**References**

1. Smith PK, Krohn RI, Hermanson GT, Mallia AK, Gartner FH, et al. (1985) Measurement of Protein Using Bicinchoninic Acid. Analytical Biochemistry 150: 76-85.
