# Supplementary material for: Promotion of Iron Oxide Reduction and Extracellular Electron Transfer in Shewanella oneidensis by DMSO
Source: PLoS One. 2013 Nov 7;8(11):e78466. doi: 10.1371/journal.pone.0078466 (PMC3820605; doi:10.1371/journal.pone.0078466)
Supplement: Figure S2 — The coordination structures of FeO(OH) with three ligands of H2O (a) and DMSO (b). Carbon atoms colored in gray, hydrogen white, oxygen red, sulfur yellow, and iron purple. (DOCX) [file pone.0078466.s002.docx]

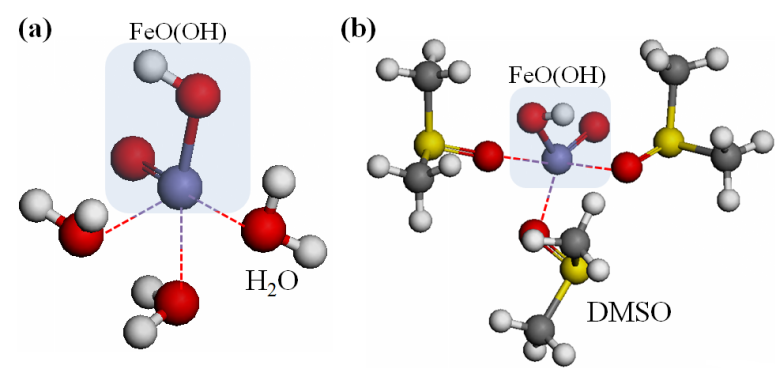


**Figure S2.** **The coordination structures of FeO(OH) with three ligands of H_2_O (a) and DMSO (b).** Carbon atoms colored in gray, hydrogen white, oxygen red, sulfur yellow, and iron purple.
