# Supplementary material for: Promotion of Iron Oxide Reduction and Extracellular Electron Transfer in Shewanella oneidensis by DMSO
Source: PLoS One. 2013 Nov 7;8(11):e78466. doi: 10.1371/journal.pone.0078466 (PMC3820605; doi:10.1371/journal.pone.0078466)
Supplement: Figure S3 — Reduction of DMSO and production of DMS by S. oneidensis MR-1 strains. DMSO was dosed at 2 mM for reduction. Samples at indicated time points from liquid and gaseous phases were collected for DMSO and DMS analysis respectively. DMS concentration was measured using a gas chromatographysystem (GC7890A, Agilent Co., USA) with a flame ionization detector. Gas was sampled from headspace of serum bottles with a syringe and directly injected into gas chromatography for analysis. A commercial GC capillary column (DB-FFAP, 30 m×0.25 mm×0.25 µm, J&W Scientific Inc., USA) was used for separation. Nitrogen (99.999%) was used as the carrier gas. The temperatures of the injector and detector were set at 250°C and 300°C, respectively. The oven temperature profile was programmed as follows: 70°C held for 3 min and ramped to 200°C at 20°C/min held for 3 min. (DOCX) [file pone.0078466.s003.docx]

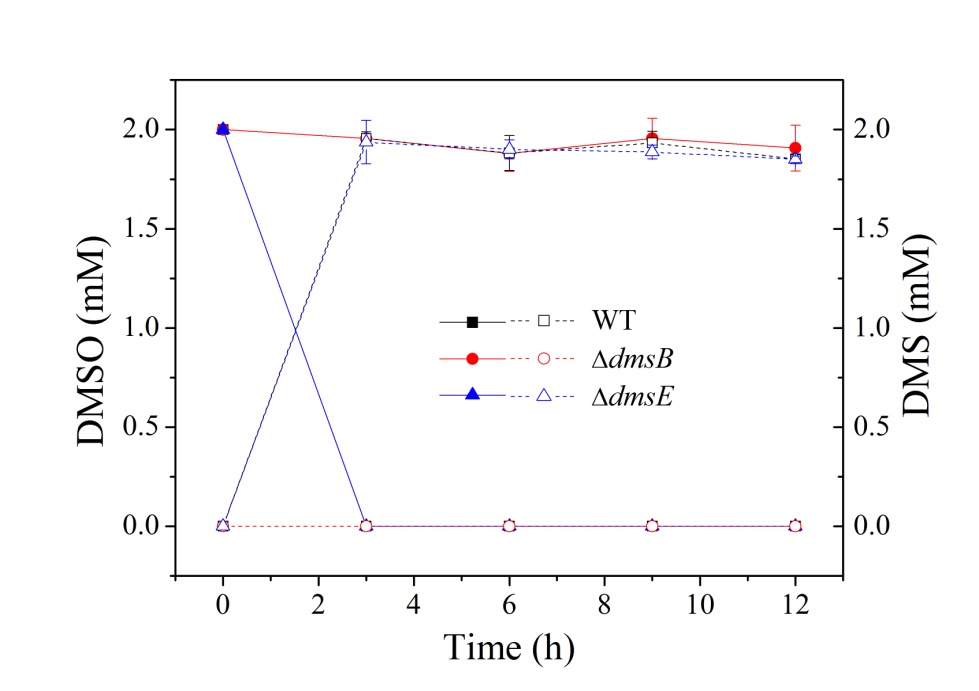


**Figure S3. Reduction of DMSO and production of DMS by *S. oneidensis* MR-1 strains.** DMSO was dosed at 2 mM for reduction. Samples at indicated time points from liquid and gaseous phases were removed for DMSO and DMS analysis, respectively. DMS measurements were conducted on a gas chromatography (GC) system (GC7890A, Agilent, USA) with a flame ionization detector (FID). Gas was sampled from headspace of serum bottles with a syringe and directly injected into GC for analysis. A commercial GC capillary column (HP-PLOT Q, 30 m × 0.53 mm × 0.40 μm, J&W Scientific, USA) was used for separation. Nitrogen (99.999%) was used as the carrier gas. The temperatures of the injector and detector were set at 250 °C and 300 °C, respectively. The oven temperature profile was programmed as follows: 60 °C held for 2 min and ramped to 240 °C at 30 h°C/min held for 3 min.
