# Supplementary material for: Promotion of Iron Oxide Reduction and Extracellular Electron Transfer in Shewanella oneidensis by DMSO
Source: PLoS One. 2013 Nov 7;8(11):e78466. doi: 10.1371/journal.pone.0078466 (PMC3820605; doi:10.1371/journal.pone.0078466)
Supplement: Figure S4 — Effect of DMS on HFO reduction by S oneidensis MR-1. 20 mM DMS was dosed into serum vials in HFO reduction. Control was set as no cells to evaluate possible abiotic reduction of HFO by DMS. (DOCX) [file pone.0078466.s004.docx]

**Figure S4. Effect of DMS on HFO reduction by *S oneidensis* MR-1.** 20 mM DMS was dosed into serum vials during HFO reduction. Control was set as no cells to evaluate possible abiotic reduction of HFO by DMS.
