# Supplementary material for: Promotion of Iron Oxide Reduction and Extracellular Electron Transfer in Shewanella oneidensis by DMSO
Source: PLoS One. 2013 Nov 7;8(11):e78466. doi: 10.1371/journal.pone.0078466 (PMC3820605; doi:10.1371/journal.pone.0078466)
Supplement: Figure S5 — Effect of DMS on electricity generated by the WT. Bacterial cells were inoculated to the OD600 of 0.5. The potential of working electrodes was set at +0.15 V (versus Ag/AgCl). DMSO and DMS were dosed at 0.5 mM. Electrochemical cells dosing DMSO or DMS and without cultures were set as controls. The experiments were repeated twice. (DOCX) [file pone.0078466.s005.docx]

**Figure S5**. **Effect of DMS on electricity currents generated by the WT.** Bacterial cells were inoculated to the OD_600_ of 0.5. The potential of working electrodes was set at +0.15 V (versus Ag/AgCl). DMSO and DMS were dosed at 0.5 mM. Electrochemical cells dosing DMSO or DMS and without cultures were set as controls. The experiments were conducted two times.
