# Supplementary material for: Promotion of Iron Oxide Reduction and Extracellular Electron Transfer in Shewanella oneidensis by DMSO
Source: PLoS One. 2013 Nov 7;8(11):e78466. doi: 10.1371/journal.pone.0078466 (PMC3820605; doi:10.1371/journal.pone.0078466)
Supplement: Figure S6 — Lactate consumption and acetate production at 48 h of HFO reduction. The medium was supplemented with 50 mM lactate for HFO reduction. 20 mM DMSO was also added when indicated. (DOCX) [file pone.0078466.s006.docx]

**Figure S6**. **Lactate consumption and acetate production at 48 h of HFO reduction.** The medium was supplemented with 50 mM lactate for HFO reduction. 20 mM DMSO was also added when indicated.
